# Human papillomavirus infection in women who develop high-grade cervical intraepithelial neoplasia or cervical cancer: a case–control study in the UK

**DOI:** 10.1038/sj.bjc.6602538

**Published:** 2005-04-12

**Authors:** M J Grainge, R Seth, C Coupland, L Guo, T Rittman, P Vryenhoef, J Johnson, D Jenkins, K R Neal

**Affiliations:** 1 Division of Epidemiology and Public Health, School of Community Health Sciences, University of Nottingham Medical School, Nottingham NG7 2UH, UK; 2 Division of Pathology, University of Nottingham Medical School, Nottingham NG7 2UH, UK; 3 Division of Primary Care, University of Nottingham Medical School, Nottingham NG7 2UH, UK; 4 Division of Cellular Pathology, Nottingham City Hospital, Nottingham NG5 1PB, UK

**Keywords:** human papillomavirus, cervical neoplasia, case–control study, older women, United Kingdom

## Abstract

Human papillomavirus (HPV) testing might identify older women who could be withdrawn from the cervical screening programme, or require less frequent screening. A case–control study using the United Kingdom cervical screening population was set up to help address this issue. Cases comprised 575 women who developed cervical intraepithelial neoplasia (CIN) grade 2 or worse over a 13-year period following a cytologically normal baseline smear, and were stratified by age group (‘under 20’, ‘20–39’ and 40 years or over). Controls (*n*=601) were women who remained disease free over this interval and were the same age on average as cases. DNA was extracted from the baseline smears and tested for HPV by PCR using GP5+/6+ consensus primers. HPV+ samples were tested for HPV types 16 and 18 using specific PCR primers. In all, 27.0% of cases tested positive for HPV at baseline, compared with 15.4% of controls (odds ratio (OR)=2.00; 95% confidence interval (CI), 1.50–2.68). Among women aged 40 years or over, the OR for HPV 16 was 8.95 (95% CI, 2.63–30.4). These results support the need for further cervical screening of HPV− older women, as many of the cases were HPV− at baseline.

Human papillomavirus (HPV) infection has been found to be present in 99.7% of cervical cancers, and is thus acknowledged as a necessary cause of this disease (Walboomers *et al*, 1999). High-risk viral types have been found to have odds ratios (ORs) in excess of 150 for cervical cancer when HPV status was ascertained at the time of cancer diagnosis (Munoz *et al*, 2003). To establish the extent to which a test for HPV can predict future cervical cancer or high-grade cervical intraepithelial neoplasia (CIN), either prospective (Koutsky *et al*, 1992; Schlecht *et al*, 2003) or nested case–control studies are needed (Liaw *et al*, 1999; Wallin *et al*, 1999; Ylitalo *et al*, 2000; van der Graaf *et al*, 2002).

In case–control studies where archived cervical smears have been used to detect past infection with genital HPV types, HPV infection has been found to be associated with both subsequent invasive cervical cancer and carcinoma *in situ* (Wallin *et al*, 1999; Ylitalo *et al*, 2000). Equivalent data both for a United Kingdom population and among older women are required as it has been suggested that some HPV− older women could be safely withdrawn from the cervical screening programme in the UK before the current cessation age of 65 years (Sherlaw-Johnson *et al*, 1999).

The aim of the present work was to examine the association between HPV and subsequent cervical disease through a case–control study, where the case group consisted of a large number of UK women who developed CIN grade 2 or worse after a cytologically normal baseline smear. A substantial number of these were aged 40 years or over at baseline.

## MATERIALS AND METHODS

### Selection of cases

Cases were identified using cytology and histology records from the Nottingham cervical cytology centre. Our primary interest was women aged 40 years or over who had a cytologically normal screening smear taken between May 1988 and November 1992 (termed the baseline smear) and who subsequently received a histologically confirmed diagnosis of CIN grades 2 or 3 or cervical cancer up until September 2002. In the UK, cervical smears have to be retained for 10 years for legal reasons, meaning that cervical smears taken after 1992 could not be retrieved for HPV testing. In total, 204 cases aged 40 years or over at baseline were identified as eligible and had baseline smears that could be retrieved. Women aged under 40 years at baseline were also recruited to increase the overall sample size of the study and to test the hypothesis that the relationship between the HPV measures and outcome was modified by age (although prestudy sample size calculations were not based on this hypothesis; hence, we acknowledge that these interaction tests may have lacked statistical power). The younger cases were selected consecutively on the basis of the date of their baseline smear beginning from May 1988 until around the end of 1988, in order to achieve a sample size of 100 cases aged under 20 years and 275 cases aged 20–39 years. For all age groups, the earliest smear was chosen when more than one was available (between May 1988 and November 1992) from each woman in the study, so as to maximise the length of follow-up available. Any women who had an abnormal cervical smear of mild severity or worse prior to the baseline smear were excluded from the list of possible cases. If a smear of ‘borderline’ severity was recorded prior to baseline, the woman would be eligible if this was followed by two cytologically normal smears (including the baseline smear).

### Selection of controls

Controls for this study were frequency matched by 10-year age band (under 20, 20–29, 30–39, 40–49, 50–59, 60 years or over) and year of baseline smear, in a ratio to cases of 1 : 1. The eligibility criteria for the control group were a normal baseline smear and no subsequent development of abnormal cytology (mild severity or worse) or diagnosis of CIN 2/3 or invasive cancer up until September 2002 (the time at which the case group was selected). Women with a smear recorded as having borderline dyskaryosis during follow-up were eligible as controls if this was followed by two normal smears. Selection as a control was not conditional on having a complete history of normal smears over the subsequent 10–13 years, as the purpose was to select a group of women representative of all women who did not develop cervical disease over this time period. However, all controls were required to have had a minimum of one follow-up smear in order to limit the potential for the control group to have contained women who received a diagnosis of CIN 2 or worse outside of the Nottingham area. Exclusion criteria for controls on the basis of smear abnormalities prior to baseline were identical to those for cases.

Controls were selected at random from all those eligible, ensuring that the number of cases and controls were equal within each age band and for each year of baseline smear. At the time of slide retrieval, replacement of damaged or missing smears was carried out for controls by randomly selecting a smear from another woman on the list of eligible controls. There was a slight imbalance in the number of cases and controls aged 40 years or over (*n*=224 controls *vs n*=204 cases) due to the fact that replacement smears were available for controls but not for cases.

### Anonymisation and ethical considerations

The Nottingham City Hospital Ethics Committee agreed that women selected for this study would not be contacted in order to provide consent. This was decided in the context of the 1999 ‘Consensus Statement of Recommended Policies for Uses of Human Tissue in Research Education and Quality Control’ (Royal College of Pathologists, 1999). When cytology slides were retrieved, identifiers were erased and replaced with a unique study number. The study was anonymised so that only investigators based at the cytology centre (PV, JJ) had access to full clinical data on the study participants; these investigators did not have access to HPV testing results at an individual level. Laboratory personnel had no information on the study participants other than the above study number, and were blinded to case–control status.

### HPV testing

Cytology slides were immersed in xylene (40 ml) for 2–3 days to remove coverslips. Cells were then scraped into an Eppendorf tube containing ethanol (1 ml) using a sterile scalpel blade and centrifuged to remove any traces of xylene. DNA was extracted using Qiagen™ extraction kits (Qiagen, 2004a).

*β*-globin PCR was carried out for 354 samples (a mixture of cases and controls) in order to test the integrity of the DNA extraction process, of which 342 samples (96.6%) tested positive. Owing to the high percentage of women with *β*-globin+ smears and the need to preserve the DNA sample, it was decided that no further *β*-globin PCR's would be performed.

HPV DNA was amplified using real-time PCR (Stratagene Mx4000) (Seth *et al*, 2004). The GP5+ and GP6+ consensus primer pair located in the L1 region of the HPV genome was used here. These primers amplify a broad spectrum of genital HPV types in a single reaction (including presently unsequenced HPV types); hence, infections with both high- and low-risk HPV types were detected (de Roda Husman *et al*, 1995). The PCR master mix (Quantitect, purchased from Qiagen, 2004b) contained optimised amounts of SYBR® Green dye to which primers (5 pmol tube^−1^) and DNA template (5 *μ*l) were added. Standard tubes of HPV 16 DNA (0.01–10 pg tube^−1^) and blank tubes (no DNA) were included in all assays. In all, 40 amplification cycles were performed. Samples that were HPV+ using the GP5+/6+ primer sequence were also tested for HPV 16 and HPV 18 using type-specific PCR primers located within the E7 gene.

### Statistical analysis

Odds ratios and 95% confidence intervals (CIs) were calculated using logistic regression analysis adjusted for age at baseline. Age was fitted as a linear term for these analyses, although results were similar when models were rerun with age as a categorical variable. Subgroup analyses were carried out for age group (under 20, 20–39 and 40 years or over), and with the case group stratified by both grade of diagnosis (CIN 2, CIN 3 or cervical cancer including microinvasive squamous cell carcinoma (SCC)) and time in years between baseline and diagnosis (<4, 4–8 and >8 years). Exposure measures were infection with any genital HPV type (based on results from the GP5+/6+ assay) and infection with HPV types 16 and 18 specifically.

## RESULTS

### Characteristics of sample

A total of 1176 women were selected for this study (575 cases and 601 controls). Four cases were excluded when a review of the biopsy report found that the outcome lesion did not conform to a grading of CIN 2 or worse. There were insufficient cells for DNA extraction for 17 smears (seven cases and 10 controls); hence, these women were excluded. This left a final sample size of 564 cases and 591 controls. The numbers of cases and controls by age group are shown in Table 1. Of the cases, 167 (29.6%) women had a diagnosis of CIN 2, 346 (61.3%) had a diagnosis of CIN 3 and 51 (9.0%) had developed cervical cancer (20 with adenocarcinoma, 16 with SCC, nine with microinvasive SCC and six with adenocarcinoma *in situ*). Among cases, the median length of time between the baseline smear and diagnosis of outcome was 6.8 years (range=0.1–13.5 years) and the median age at diagnosis was 36.2 years (range 19.1–76.1 years). Of the four cancer cases who were under age 20 years at baseline, all developed the outcome 9 or more years after the time of their baseline smear (minimum age at diagnosis=27 years). Exclusion of four cases where the time between baseline and diagnosis was less than 6 months had little impact on results presented below.

### Overall HPV rates

A comparison of the rates of infection with any genital HPV type (high or low risk) between cases and controls both for the entire sample and among specific age groups is provided in Table 2. Cases had significantly higher rates of HPV infection in their baseline smears than controls (27.0 *vs* 15.4%). Overall, HPV+ women had a two-fold increase in the risk of developing an outcome lesion. The OR was highest for women aged 40 years or older at baseline, while for the smaller group of women under age 20 years, the difference in HPV status between cases and controls was not statistically significant. A test for interaction to compare the magnitude of the ORs across the three age groups (with age group fitted as a linear term) failed to reach statistical significance (*P*=0.15).

### Stratification by grade and time

Cases were stratified according to grade of outcome (or worse outcome for cases who had more than one abnormal biopsy during the follow-up period) and the length of time between the baseline smear and diagnosis; these results are presented in Table 3. The risk of a more severe grade of outcome lesion increased with baseline HPV infection, with a significant trend across the case groups. This trend was most obvious among women aged 20–39 years, among whom eight out of 14 (57.1%) women with cancer tested positive for HPV at baseline (data not shown). The rate of baseline HPV positivity was also highest for cases diagnosed within 4 years of the negative baseline smear, again with a significant linear trend. When these two stratification variables were considered jointly, the OR was highest for cancer cases diagnosed between 4 and 8 years after the baseline smear, although because of the overlapping confidence intervals no definite conclusions can be made about the relative effect sizes for the nine case groups (Table 3).

### Rates of HPV 16 and 18

A total of 70 cases and 18 controls tested positive for HPV 16 and 27 cases and 17 controls were positive for HPV 18. These results are described in more detail in Table 4. Odds ratios were higher for exposure to HPV 16 compared with exposure to any HPV type within all three age categories (comparison with results in Table 2). The association remains nonsignificant, however, for the under 20 years age group. An interaction term fitted to compare the ORs by age group (with age group fitted as a linear term) failed to reach statistical significance (*P*=0.086). HPV 18 does not have a significant effect upon the odds of being a case, despite raised ORs for all three age groups. The interaction between age group and HPV 18 was also nonsignificant (*P*=0.77). When additional models were fitted to control for infection with the other high-risk HPV type (16 or 18) and for other HPV types (positive using GP5+/6+ primers but negative for HPV 16/18), the ORs remained significant for HPV 16 and nonsignificant for HPV 18. The interaction term with age group remained of borderline significance for HPV 16 (*P*=0.066) and nonsignificant for HPV 18 (*P*=0.67) when the other HPV terms were adjusted for. When analyses were performed separately for the three grades of cases with results combined over age groups and adjusted for the other HPV types, the effect of HPV 16 was significant among all case groups (data not shown). For HPV 18, there was a higher OR among cancer cases, although this was not statistically significant (OR=2.99; 95% confidence interval (CI), 0.94–9.49; *P*=0.063).

## DISCUSSION

This study found that HPV significantly predicts the development of high-grade cervical disease an average of 6.8 years later. The magnitude of the ORs varied according to age at baseline smear, grade of outcome, time to development of outcome and HPV type. For women aged 40 years or over, infection with HPV 16 was associated with an approximate 10-fold increase in risk of CIN 2 or worse. Despite this, however, 73% of cases were HPV− at baseline. Strengths of the study include the large number of cases, the long period of follow-up and relevance of the study sample to a population where HPV testing could be introduced in practice (older women based in the United Kingdom).

Several aspects of the study design merit attention. First, controls were not individually matched to cases, instead it was ensured that numbers of cases and controls were equal within categories defined by age (10 year bands) and year of baseline smear. At the time of inception of this study, there was some concern that many archival cervical smears would contain insufficient DNA for HPV analysis (as indicated by results from the *β*-globin assay). The conditional logistic regression method of analysis needed for matched case–control data requires data to be nonmissing for both case and control(s); hence, a large number of missing values for the exposure variable would make such an analysis highly inappropriate. All logistic regression analyses presented here were adjusted for age to allow for the fact that within each age band, the age distribution of cases was not always uniform, whereas it was for controls. Second, the selection criteria for our control group was for women to have had just one subsequent normal cervical smear, as opposed to a complete history of normal smears over the 10–13 year follow-up period. We cannot therefore be sure that all controls remained disease free over this period, as they may have migrated and presented with CIN outside of Nottingham. Among a retrospective cohort of women aged 40 years or over participating in the Nottingham screening programme, less than 1 woman per 1000 was diagnosed with CIN (grades 2 or 3) or cancer each year (unpublished data). This would therefore have had a negligible impact on our results, although we acknowledge the possibility that such misclassification could have been greater for the younger age groups, among whom the incidence of CIN is higher.

Data are available to suggest that among women who develop cervical cancer, the high rate of false cytological negativity among previous cervical smears is an issue (Walboomers *et al*, 1995). In this study, smears from cases and controls were not reviewed to confirm that they were cytologically normal. It can be argued that as the primary intention of this study was to estimate the impact of HPV testing within a screening programme, the inclusion of false negatives among the baseline smears would reflect clinical practice, and hence their exclusion would be unadvisable (Woodman and Collins, 2002). Furthermore, in a previous case–control study of cervical cancer, the percentage of cases who were HPV+ at baseline was very similar among women whose smears were reclassified as abnormal (69%) compared with those whose smears remained normal (70%); therefore, the impact on results of excluding the former group would be negligible (Zielinski *et al*, 2001).

Prospective and nested case–control studies of HPV and subsequent CIN/cancer have reported ORs of differing sizes (Woodman and Collins, 2002). Factors that are likely to affect the size of an observed OR include, among others, the average length of time between the baseline smear and diagnosis of outcome and the HPV types tested for (all genital types or high risk types only). Studies where results have been stratified by the former have reported higher ORs when the time to diagnosis is shorter (Wallin *et al*, 1999; Ylitalo *et al*, 2000). This may be consistent with our finding of higher ORs among women aged 40 years or over, among whom the incidence of new HPV infections after baseline is likely to be lower. Our overall OR (obtained using the GP5+/6+ primer sequences) was lower than for many other studies (Wallin *et al*, 1999; Carozzi *et al*, 2000; van der Graaf *et al*, 2002), which must in part be due to the fact that the PCR primers used here detected a combination of high- and low-risk HPV types. Data from other follow-up studies have shown that high-risk HPV types are associated with a higher risk of subsequent CIN/cancer than low-risk types (Liaw *et al*, 1999; Kjaer *et al*, 2002; van der Graaf *et al*, 2002). Our OR for the high-risk type HPV 16 was similar to that found in a Swedish study, which also used archived cervical smears (ORs ranged from 5 to 7 depending on age at baseline), where HPV 16 exposure was assessed in smears taken an average of 7.8 years before diagnosis (Ylitalo *et al*, 2000). It was also consistent with a study of women aged 50 years or over from the United Kingdom screening population, where the OR for HPV type 16 was 10.3, where the outcome was a subsequent abnormal smear over a 10-year follow-up period (Cruickshank *et al*, 2002). Two other studies, however, have reported ORs for HPV type 16, which were noticeably higher than those observed here (Liaw *et al*, 1999; van der Graaf *et al*, 2002).

Another possible explanation for the lower ORs observed here, both for overall HPV and specifically for HPV 16, is the fact that long-stored archival material was used in the laboratory analysis. Other studies that have used archival cervical smears have reported both differing ORs and differences in the percentage of the case group who were positive for HPV at baseline (Wallin *et al*, 1999; Carozzi *et al*, 2000; Ylitalo *et al*, 2000; van der Graaf *et al*, 2002). In our study, the number of cells recovered was small in many instances, this could result in the possibility of false-negative HPV results, which if occurring equally for case and control subjects would bias the observed ORs towards unity. This would not, however, provide an explanation for the relatively high rate of HPV positivity observed among control women (10% in controls aged 40 years or over). A difference in the sensitivity of the HPV test favouring controls would be counterintuitive, on the basis that replication of the HPV virus should be higher in women who subsequently go on to develop cervical disease (cases). The alternative explanation is that crosscontamination of DNA samples resulted in increased rates of HPV positivity among controls. This could potentially occur either at the time of smear collection at GP practices, at the screening laboratory during the process of fixation and staining, or in the HPV testing laboratory. While set procedures in our laboratory were followed to ensure that no contamination occurred at the stage of HPV testing, contamination at the time of sample collection or preparation has been recognised as a potential problem when studying archived material (Chua and Hjerpe, 1995). Such crosscontamination of DNA samples affecting cases and controls in equal measure would have the effect of lowering observed ORs. Although there is evidence that archival smears are a reliable resource for detecting past HPV infection (Jacobs *et al*, 2000), there is a shortage of data where HPV results from the same woman (including typing) are compared between fresh and long-stored archived cervical smears. Overall therefore, further research is still needed to determine the impact of using long-stored specimens for epidemiological purposes such as those described here.

The causal relationship between HPV and subsequent CIN/cancer is now well established, but this alone does not provide sufficient evidence for the introduction of HPV testing in the cervical screening of older women. In our study, over 70% of cases tested negative for HPV at baseline. This percentage was higher among women where the time between baseline and diagnosis was more than 8 years, but was still 67% among those who developed CIN/cancer within 4 years of baseline; however, for cases aged 40 years or over, 75% were HPV− at baseline. In a case–control from Sweden where invasive cancer was the outcome and the average age at baseline was 44 years, just four out of 35 women (11.4%) who developed cancer more than 6 years after baseline were positive for HPV using a consensus PCR method (MY09/11) (Wallin *et al*, 1999). These results taken in conjunction would not support the withdrawal of HPV− older women from cervical screening, but need to be interpreted in light of the fact that use of stored (archived) specimens may have resulted in an underestimation of HPV infection rates at baseline due to inadequate DNA preservation. In the United Kingdom, it has been suggested that cessation of screening for low-risk older women should take place at age 50 (Flannelly *et al*, 2004). A direct estimate of the negative predictive value of an HPV test at or shortly after the age of 50 years can only be obtained from a large cohort study where fresh HPV samples are obtained from participants at study entry. Such data may be required before it can be ascertained for certain whether or not HPV− women could be safely withdrawn from cervical screening in the UK.

## Figures and Tables

**Table 1:** No. of cases and controls by age at baseline smear

**Baseline age (years)**	**Cases (total)**	**CIN 2**	**CIN 3**	**Cancer**	**Controls**
Under 20	97	30	63	4	100
20–39	267	80	173	14	269
40 or over	200	57	110	33	222

Total	564	167	346	51	591

CIN=cervical intraepithelial neoplasia.

**Table 2:** No. (%) of cases and controls positive for any genital HPV type by age at baseline, along with results from logistic regression analysis

	**Cases**	**Controls**	
	**HPV+**	**HPV+**	
**Age group (years) (baseline)**	**N (%)**	**N (%)**	**OR^a^ (95% CI)**
Under 20	24 (24.7)	18 (18.0)	1.62 (0.80–3.28)
20–39	79 (29.6)	50 (18.6)	1.82 (1.21–2.72)
40 or over	49 (24.5)	23 (10.4)	2.79 (1.63–4.79)

All ages	152 (27.0)	91 (15.4)	2.00 (1.50–2.68)

HPV=human papillomavirus; OR=odds ratio; CI=confidence interval.

a Adjusted for age in years using logistic regression.

**Table 3:** ORs^a^ showing the effect of HPV positivity at baseline, with cases stratified jointly by grade of diagnosis and time to diagnosis

	**Time to diagnosis**				
	**⩾8 years (*n*=209)**	**4–8 years (*n*=205)**	**<4 years (*n*=150)**	**All cases (*n*=564)**	**Linear trend^b^**
*Grade of diagnosis*					
CIN 2 (*n*=167)	0.93 (0.44–1.99)	1.56 (0.81–3.03)	2.30 (1.23–4.31)	1.55 (1.01–2.38)	*P*=0.022
	(9/55)	(13/57)	(16/55)	(38/167)	
CIN 3 (*n*=346)	1.55 (0.97–2.47)	1.90 (1.21–2.98)	3.82 (2.30–6.33)	2.13 (1.54–2.95)	*P*<0.001
	(31/132)	(35/133)	(32/81)	(98/346)	
Cancer (*n*=51)	2.83 (1.11–7.23)	5.65 (1.95–16.3)	1.38 (0.28–6.81)	3.32 (1.71–6.48)	*P*<0.001
	(7/22)	(7/15)	(2/14)	(16/51)	
All cases (*n*=564)	1.47 (0.99–2.20)	1.97 (1.34–2.89)	2.97 (1.96–4.50)	—	*P*<0.001
	(47/209)	(55/205)	(50/150)		

Linear trend^c^	*P*=0.029	*P*<0.001	*P*<0.001	*P*<0.001	

OR=odds ratio; HPV=human papillomavirus; CIN=cervical intraepithelial neoplasia.

Numbers in cells represent the OR for the case group compared with controls (and 95% CI), the numbers within parentheses underneath are the number of HPV+ women/total number of women in case group.

a All ORs are adjusted for age using logistic regression.

b The linear trend was calculated by fitting time to diagnosis as the outcome variable in an ordinal logistic regression model (with controls comprising the baseline group).

c The linear trend was calculated by fitting grade of lesion as the outcome variable in an ordinal logistic regression model (with controls comprising the baseline group).

**Table 4:** No. (%) of cases and controls positive for HPV 16 and HPV 18 along with results from logistic regression analysis

	**Cases** **HPV+**	**Controls** **HPV+**	**OR^a^ (95% CI)**	
**Age group at baseline**	***N* (%)**	***N* (%)**	**Model 1^b^**	**Model 2^c^**
*HPV 16*				
Under 20	9 (9.3)	5 (5.0)	2.25 (0.71–7.13)	2.10 (0.61–7.30)
20–39	39 (14.6)	10 (3.8)	4.62 (2.24–9.51)	4.15 (1.99–8.64)
40 or over	22 (11.0)	3 (1.4)	8.95 (2.63–30.4)	9.08 (2.65–31.1)
All ages	70 (12.4)	18 (3.1)	4.48 (2.61–7.55)	4.18 (2.44–7.18)

*HPV 18*				
Under 20	3 (3.1)	3 (3.0)	1.12 (0.22–5.78)	0.85 (0.15–4.95)
20–39	12 (4.6)	6 (2.3)	2.02 (0.74–5.49)	2.15 (0.72–6.44)
40 or over	12 (6.0)	8 (3.6)	1.69 (0.68–4.23)	1.30 (0.49–3.48)
All ages	27 (4.8)	17 (2.9)	1.72 (0.93–3.20)	1.48 (0.77–2.86)

HPV=human papillomavirus; OR=odds ratio; CI=confidence interval.

a For cases *vs* controls (logistic regression).

b Adjusted for age in years.

c Adjusted for age in years, infection with other specific HPV type (16 or 18) and infection with unknown HPV type (positive using GP5+/6+ primers but negative for HPV 16/18).
